# Case report: An intrauterine device hugging the musculus rectus abdominis through the center of a cesarean scar

**DOI:** 10.3389/fsurg.2022.956856

**Published:** 2023-01-06

**Authors:** Jigang Jing

**Affiliations:** Department of Ultrasound Medicine, West China Hospital, Sichuan University, Chengdu, China

**Keywords:** intrauterine device (IUD), ultrasound, computed tomography, migration, MCu-IUD, case report

## Abstract

A 34-year-old woman presented with an abscess of the abdominal wall, with a 10-day history of rupture and discharge. She had a history of cesarean section 17 years ago, intrauterine device (IUD) insertion 5 years ago, and intermittent lumbago and abdominal pain for the past 4 years. A V-shaped IUD was observed via ultrasound between the bladder and abdominal wall, clinging to the musculus rectus abdominis through the center of the cesarean scar. Pelvic computed tomography (CT) revealed a V-shaped metal density at the anterior upper edge of the bladder, one end of which seemed to penetrate the abdominal wall. IUD migration was confirmed by surgery 2 days later. The patient recovered after IUD removal. This case affirms that a migrated IUD can lead to serious complications, even if there are no obvious symptoms for many years. Apart from the fact that the IUD has fallen out of place, a migrated IUD should be removed promptly, regardless of obvious complications. Furthermore, regular ultrasound examination is important for early diagnosis of IUD displacement. Based on the relevant literature, we also hypothesize the possible mechanism of IUD migration between the anterior bladder wall and the abdominal wall.

**Core tip:**

Uterine perforation and IUD migration to the organs in the abdominopelvic cavity are serious complications of IUD insertion. We present a case of uterine perforation complicated by IUD migration with the application of intraoperative ultrasound localization. This case highlights that ultrasound, especially intraoperative ultrasound, can provide objective information for the diagnosis and localization of IUD migration, with the advantages of point of care, real-time imaging, convenience, low cost, and lack of radiation. Based on this case and on the relevant literature, we hypothesized the possible mechanism of IUD migration between the anterior bladder wall and the abdominal wall. To the best of our knowledge, no previous research has discussed the process of IUD migration beyond the anterior wall of the bladder.

## Introduction

Intrauterine devices (IUDs) are commonly used contraceptives in clinical practice, and they have been endorsed as first-line choices for nulliparous and parous adolescents (1). IUD complications include hemorrhage, uterine perforation, infection, ectopic migration, rupture, deformation, dislocation, and downward movement (2). Down-migration is the most common of these complications, but uterine perforation is among the most serious. The incidence of complete or partial uterine perforation was reported to be 1.6 per 1,000 insertions (3).

Once the uterus is perforated, the IUD can move within the ventral pelvic cavity. Although the IUD can migrate to any organ, many cases reported in recent years have found migration to the intestinal tract (4–6), urinary tract (7), omentum (8), and intrathoracic cavity (9). Uterine perforation and IUD migration are critical complications of IUD insertion and can be successfully treated by laparoscopy, or by laparotomy in cases of severe pelvic adhesion or unforeseen complications (10, 11). In addition to gynecological examination, abdominal ultrasound, transvaginal ultrasound, 3D ultrasound, and CT have been used to diagnose and locate migrated IUDs (12). The use of intraoperative ultrasound has been adopted in the removal of migrated IUDs (13). However, while reports of IUD migration have increased, migration beyond the anterior bladder wall has rarely been studied; to the best of our knowledge, no previous research has described this specific migration process. In this report, we present a rare case of IUD migration between the anterior bladder wall and a cesarean section scar.

### Case report

A 34-year-old woman was admitted to the hospital because of recurrent lumbago and abdominal pain for 4 years, aggravated by subumbilical abdominal wall discharge for 10 days. She had undergone a cesarean section 17 years ago and an IUD insertion 5 years ago. There was no obvious cause for the lumbago and abdominal pain. It was accompanied by acid reflux and bloating and persistent dull pain; the patient did not complain of radiating pain, fever, coughing, headache, flustered or tiredness, or edema of the lower limbs. These symptoms sometimes got better and sometimes got worse; she therefore did not seek medical treatment. Ten days prior to admission, a metal foreign body was found in the skin of the abdominal wall, surrounded by redness and pain, with pus discharging around it. The patient’s vitals were as follows: Temperature: 36.5°C, Pulse: 73 b/m, Respiratory: 20 b/m, blood pressure: 129/91 mmHg, weight: 49 kg, height: 150 cm. The vital signs were normal.

**Figure 1:**
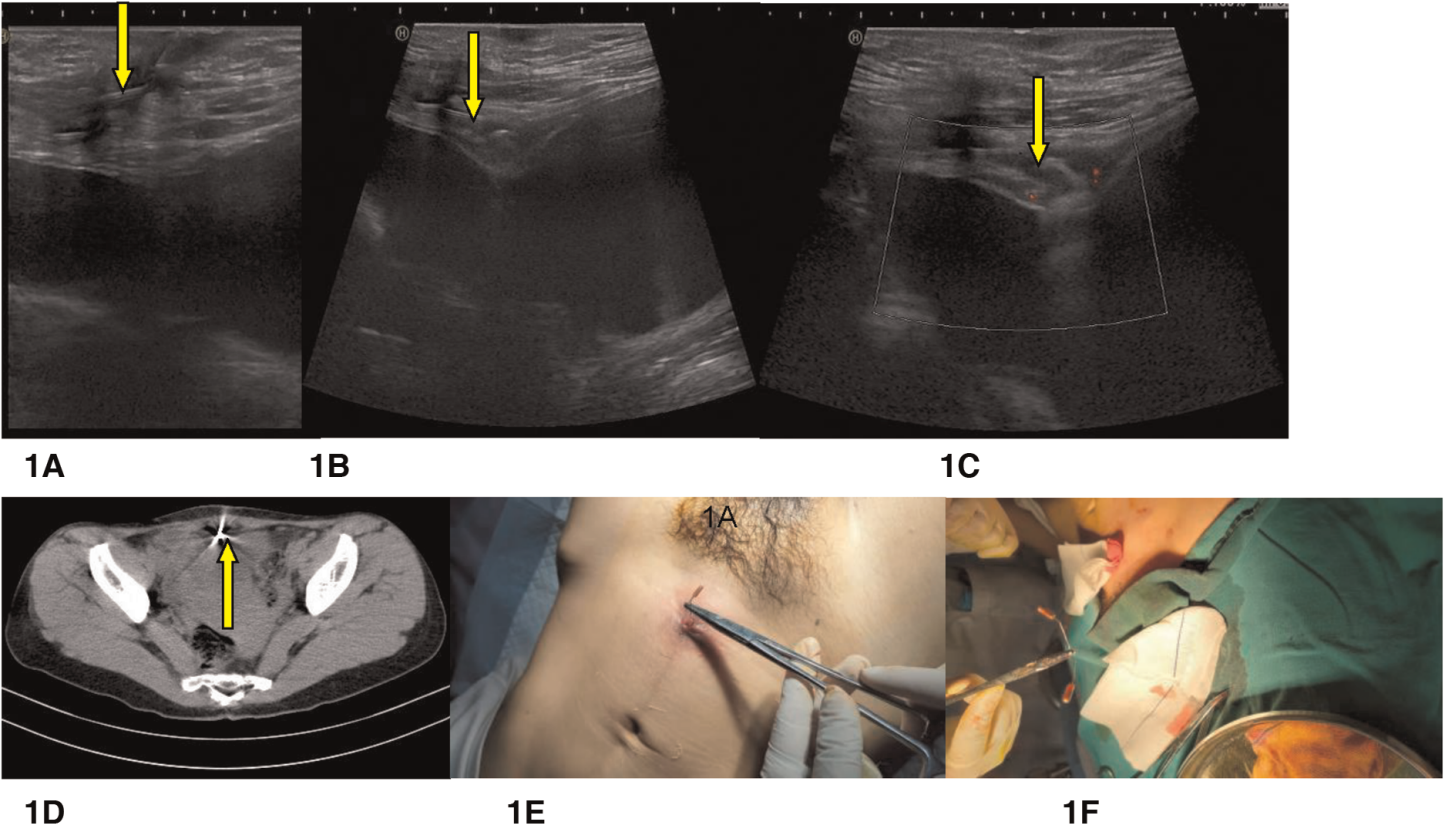
A 34-year-old woman presented with an abscess of the abdominal wall around the IUD, which had been discharging pus for 10 days. Yellow arrows indicate the IUD. (**A**) In this longitudinal section of the lower abdominal wall, linear hyperechoes (IUD) can be seen, folding the rectus abdominis through the center of the cesarean scar. (**B**) In this cross-section of the inferior abdominal wall, linear hyperechoes (IUD) can be seen, clasping the straight muscle of the abdomen through the center of the cesarean scar. (**C**) In this cross-section of the lower abdominal wall, abscess echoes can be seen in front of the anterior wall of the bladder, and dotted blood flow signals can be seen surrounding them. (**D**) CT cross-section of the inferior abdomen. A V-shaped metal IUD can be seen on the lower abdominal wall. (**E**) Part of the IUD was removed from the lower abdominal wall during the operation. (**F**) The MCu-IUD was extracted from the inferior abdominal wall during the operation. IUD: intrauterine device.

Routine physical examination revealed a 10-cm-long horizontal cesarean scar in the lower abdomen. A palpable abscess of 4 cm was detected at the center of the scar, with one end of the IUD penetrating the skin. The gynecological examination found a small amount of yellow vaginal secretion with odor; there were no strings in the cervix, and no mass in the uterus or the annexes. The results of routine blood tests, biochemical blood tests, coagulation time, human chorionic gonadotropin (hCG), a nine-item preoperative test to screen for infectious diseases, and routine urine tests were normal. On ultrasound, the posterior uterus had normal morphology, with an anterior and posterior diameter of 4.3 cm; the uterine cavity fluid was about 0.5 cm, there was no deformation or displacement of the uterine cavity, no mass or foreign body was observed, the echoes of the muscular layer were homogeneous, and the maximum thickness of the isthmus scar was about 0.6 cm. No obvious abnormality was observed in the bilateral annexes. No anechoic area was found in the pouch of Douglas. A V-shaped IUD was detected between the bladder and abdominal wall, embracing the musculus rectus abdominis through the center of the cesarean scar. The IUD was surrounded by a hypoechoic envelope of about 2.1 × 1.1 × 1.5 cm; dotted blood flow signals were visible around it (Figure 1A–C). Transvaginal ultrasound and 3D ultrasound were not performed because the IUD had been localized by abdominal ultrasound and only one IUD had been inserted, according to the surgical history. An electrocardiogram (ECG) showed sinus bradycardia. Plain pelvic radiography and cystoscopy were not performed. Pelvic CT without contrast corroborated a V-shaped metal density at the anterior upper edge of the bladder, with one end seeming to pierce through the abdominal wall. It was considered that the IUD was highly likely to be displaced, the uterus was slightly larger, and watery density was shown inside (Figure 1D).

Two days later, a bladder catheter was inserted smoothly; drainage was unobstructed, and there were no abnormal manifestations such as hematuria. Lidocaine hydrochloride was used for local infiltration anesthesia to remove the IUD. The IUD was pulled outward with homeostatic forceps under ultrasonic guidance. After pulling out the IUD for 3 cm, it became difficult to continue. The patient and her family were consulted. Following local anesthesia, a laparotomy was immediately performed. A 2 cm incision was made, centered on the IUD. The tissue around the incision was hard and brittle. Local subcutaneous fat was then excised. The other end of the IUD was enveloped by the anterior sheath of the rectus abdominis; the anterior sheath was cut apart, allowing the removal of a complete V-shaped IUD (MCu-IUD) (Figure 1E,F). An absorbable suture was used to stitch the anterior sheath layer and the skin. No particular discomfort was reported during or after the operation. Intraoperative bleeding was about 10 ml.

## Discussion

A review of the literature suggests that reports of IUD migration are increasing, with most cases occurring in the last decade (14). Bladder calculus developing over the migrated IUD is the most common presentation (15). However, to our knowledge, this is one of the few reported cases of a migrated IUD causing an anterior abdominal wall abscess (PubMed search; search terms: “IUD,” “migrated,” and “abdominal wall abscess”).

This case is a married woman of childbearing age with a history of IUD insertion 5 years prior and uterine scarring 17 years prior. The 4-year history of lumbago with abdominal pain accompanied by urinary discomfort was thought to be caused by genitourinary infection. However, ultrasound and CT revealed that the IUD had migrated in front of the anterior wall of the bladder. Moreover, there were no previous obvious complications, such as urinary tract obstruction, bladder stones, or intestinal perforation, aside from the abdominal wall abscess she presented with. No lacerations or scars were found in the uterus or bladder by ultrasound, similar to the cases reported by Chai et al. and Jievaltienė et al. (16, 17). In their cases, when the IUD migrated into the bladder or punctured its anterior wall, neither lacerations nor scars could be seen with the naked eye in the uterus or the bladder. Another fascinating element is the fact that the IUD came to “embrace” the musculus rectus abdominis. We speculate that the perforation of the uterus or bladder can be very small, due to the elasticity and tension of the IUD, so that the perforation remains invisible to the naked eye. In cases like these, the IUD often breaks through the uterus, posterior bladder wall, anterior bladder wall, and anterior abdominal wall successively without lacerations or scars (16, 17). This may explain why the IUD migrated between the bladder and abdominal wall, as well as the previous mild symptoms.

Inexperience, insertion technique, uterine states (especially lactation and postpartum), and instrumentation have all been proposed as causal factors of IUD migration (18). In this case, a scarred uterus, a V-shaped IUD (Mcu-IUD), a small cervical canal, a tilted uterus, and the fact that the patient was sexually active are potential causes of IUD migration. Persistent lower urinary tract symptoms in women with IUD should raise the suspicion of intravesical migration (19). In this case, however, the diagnosis was delayed because of mild discomfort and the lack of routine follow-up. Ultrasound-guided removal of the IUD was performed, and ultimately, laparotomy was needed to remove the MCu-IUD. Theoretically, removal of an IUD under ultrasound guidance is easier and causes less trauma than the “blind” standard technique. Through the failure of normal ultrasound guided extraction in this patient's specific case, it was found that proper training and close cooperation among health care workers can result in successful removal of displaced IUDs under ultrasound guidance without any complications. Only by separating the adhesions and dislodging the IUD from the surrounding tissues, and by dealing with the influences of fibrosis and calcification, can the IUD be removed smoothly. It is important to properly train health care workers in ultrasound-guided removal of ectopic IUDs. Symptomatic patients frequently undergo surgery, and asymptomatic patients are managed conservatively, as the risks of surgical intervention are quite high, with a high rate of complications (20). However, this case reveals that a migrated IUD can lead to serious complications, even if there are no obvious symptoms for many years. Apart from the fact that the IUD has fallen out of place, a migrated IUD should be removed promptly regardless of obvious complications. Furthermore, regular ultrasonography is an important method for early diagnosis of IUD migration.

## Conclusion

IUDs should be followed up routinely, and a displaced IUD should be removed promptly to avoid possible serious complications.

## Data Availability

The original contributions presented in the study are included in the article/Supplementary Material; further inquiries can be directed to the corresponding author.
